# Strong correlation of downregulated genes related to synaptic transmission and mitochondria in post-mortem autism cerebral cortex

**DOI:** 10.1186/s11689-018-9237-x

**Published:** 2018-06-01

**Authors:** Matthew Schwede, Shailender Nagpal, Michael J. Gandal, Neelroop N. Parikshak, Karoly Mirnics, Daniel H. Geschwind, Eric M. Morrow

**Affiliations:** 1 Department of Molecular Biology, Cell Biology and Biochemistry, and Carney Institute for Brain Science, Brown University, Providence, RI 02912 USA; 2 Department of Human Genetics, David Geffen School of Medicine, University of California, Los Angeles, Los Angeles, CA 90095 USA; 3 Program in Neurogenetics, Department of Neurology, David Geffen School of Medicine, University of California, Los Angeles, Los Angeles, CA 90095 USA; 4 Department of Psychiatry and Kennedy Center for Research on Human Development, Vanderbilt University, Nashville, TN 37203 USA; 5 Developmental Disorders Genetics Research Program, Emma Pendleton Bradley Hospital and Department of Psychiatry and Human Behavior, Alpert Medical School of Brown University, East Providence, RI 02915 USA; 6 Hassenfeld Child Health Innovation Institute, Brown University, Providence, RI 02912 USA; 7 Present address: Department of Psychiatry, Munroe-Meyer Institute, University of Nebraska Medical Center, Omaha, NE 68198 USA; 8 Laboratories for Molecular Medicine, Brown University, 70 Ship Street, Box G-E4, Providence, RI 02912 USA

**Keywords:** Autism, Human, Cortex, Post-mortem, Transcriptome

## Abstract

**Background:**

Genetic studies in autism have pinpointed a heterogeneous group of loci and genes. Further, environment may be an additional factor conferring susceptibility to autism. Transcriptome studies investigate quantitative differences in gene expression between patient-derived tissues and control. These studies may pinpoint genes relevant to pathophysiology yet circumvent the need to understand genetic architecture or gene-by-environment interactions leading to disease.

**Methods:**

We conducted alternate gene set enrichment analyses using differentially expressed genes from a previously published RNA-seq study of post-mortem autism cerebral cortex. We used three previously published microarray datasets for validation and one of the microarray datasets for additional differential expression analysis. The RNA-seq study used 26 autism and 33 control brains in differential gene expression analysis, and the largest microarray dataset contained 15 autism and 16 control post-mortem brains.

**Results:**

While performing a gene set enrichment analysis of genes differentially expressed in the RNA-seq study, we discovered that genes associated with mitochondrial function were downregulated in autism cerebral cortex, as compared to control. These genes were correlated with genes related to synaptic function. We validated these findings across the multiple microarray datasets. We also did separate differential expression and gene set enrichment analyses to confirm the importance of the mitochondrial pathway among downregulated genes in post-mortem autism cerebral cortex.

**Conclusions:**

We found that genes related to mitochondrial function were differentially expressed in autism cerebral cortex and correlated with genes related to synaptic transmission. Our principal findings replicate across all datasets investigated. Further, these findings may potentially replicate in other diseases, such as in schizophrenia.

**Electronic supplementary material:**

The online version of this article (10.1186/s11689-018-9237-x) contains supplementary material, which is available to authorized users.

## Background

Autism spectrum disorders (ASDs) constitute a heterogeneous group of neurodevelopmental disorders characterized by impaired social interaction, disrupted development of communication skills, and repetitive behaviors [1]. Over an affected individual’s lifetime, costs of care can reach about $3.2 million while the annual cost to society is an estimated $35 billion [2]. Such burdensome costs combined with new high estimates in prevalence—including numbers as high as 1 in 68 children [3]—call for a need to understand pathophysiology fully and to develop new treatments. Genetic studies in autism have pinpointed a heterogeneous group of loci and genes, largely emerging from studies of rare and/or de novo genetic variation [4–9]. Common susceptibility variants and inherited variants have been harder to identify in autism [10–13]. Further, some recent twin studies, such as a study by Hallmayer et al., have reported a more moderate genetic heritability than older studies [14]. These studies suggest a relatively lower concordance for autism between monozygotic twins (approximately 58% concordance) and a higher concordance between dizygotic twins (approximately 20%) as compared to older twin studies on autism (see [15] for a recent meta-analysis of twin studies on autism). In addition to supporting a strong role for genetics, the results of Hallmayer et al. implicate a shared twin environment, such as the in utero environment, as an additional factor that may play a role in susceptibility to autism.

Transcriptome studies in autism have investigated quantitative differences in gene expression between the mRNA samples extracted from post-mortem tissue from patient brains as compared to control brains [16–19]. One advantage of transcriptome studies is that they may pinpoint genes and molecular processes that are relevant to pathophysiology yet the approach circumvents the need to generate hypotheses about the genetic architecture or the gene-by-environment interactions leading to disease. Gene expression represents the summation between genetic burden and environmental insults or experience. In one of the largest studies to date, gene pathways involving synapses were found to be most enriched among the genes with decreased expression in autism, whereas pathways involving neuroimmune and microglial response were enriched among the genes with increased expression in autism [17]. Similar findings were noted in a more recent and larger RNA-seq study of autism cerebral cortex [19]. Interestingly, immune gene alterations had been reported previously in autism as a preliminary finding in a much smaller dataset [16].

We have conducted an alternative analysis of the transcriptome data using differentially expressed genes from an RNA-seq dataset [19] and three previously published microarray datasets [16–18]. We discovered that a gene pathway related to mitochondrial function was downregulated in autism cerebral cortex and correlated with a pathway related to synapse function. Recent independent reports have also identified downregulation of genes related to mitochondrial processes in autism post-mortem brain [20, 21]. These transcriptome data are also concordant with additional multifaceted findings that support a role for mitochondrial dysfunction in autism pathology [22, 23]. In addition, autism severity may be correlated with abnormalities in biomarkers of mitochondrial function [22], and further still, a mitochondrial signature has been seen in other neuropsychiatric conditions, such as in schizophrenia [24]. Overall, our data support a model wherein mitochondrial processes may play an important role in the primary pathophysiology and/or progression of neuropsychiatric diseases.

## Methods

### Participants

We analyzed gene expression in autism and control cerebral cortex using genes from Parikshak et al., an RNA-seq study [19], and microarray data from three other published studies [16–18]. The primary microarray dataset was from Voineagu et al. [17] and was downloaded from the Gene Expression Omnibus (GEO, GSE28521) [25]. We limited the Voineagu et al. samples to those with information on RNA integrity number (RIN) and post-mortem interval (PMI). This dataset consisted of prefrontal and/or superior temporal gyrus samples from 15 autism and 16 control subjects (*n* = 29 control samples, *n* = 27 autism samples). The Voineagu et al. dataset also contained samples from the cerebellum, which were not used in our study. The two other microarray datasets were from Chow et al. (dorsolateral prefrontal cortex, *n* = 18 control samples, *n* = 15 autism samples) [18] and Garbett et al. (superior temporal gyrus, *n* = 6 control samples, *n* = 6 autism samples) [16]. We downloaded the Chow et al. dataset from GEO (GSE28475), and the Garbett et al. authors sent us their dataset directly. A Venn diagram depicting overlap of subjects among these datasets is shown in Additional file 1: Figure S1. See Additional file 2: Table S1 for more details about each study used for these analyses.

### Sample preparation and hybridization

The Parikshak et al. dataset samples came from the National Institute of Child Health and Human Development-funded University of Maryland Brain and Tissue Bank and the Autism Tissue Program [19]. The Voineagu et al. dataset samples came from the Autism Tissue Program and the Harvard Brain Bank [17]. The Garbett et al. dataset samples also came from the Autism Tissue Program [16], and the Chow et al. dataset samples came from the National Institute of Child Health and Human Development-funded University of Maryland Brain and Tissue Bank and the Autism Tissue Program [18]. In all studies, RNA was extracted from frozen samples. For the Parikshak et al. study, RNA sequencing was performed using an Illumina HiSeq 2000 or 2500 machine, with reads mapped to hg19 using Gencode v18 annotations. For the Voineagu et al. and Chow et al. studies, RNA was hybridized to the Illumina HumanRef8v3 microarray, which contains 24,526 probes. For the Garbett et al. study, RNA was hybridized to Affymetrix Human Genome 133 plus 2 microarrays, which has 54,675 probe sets.

### Data normalization and characteristics

Each microarray dataset was downloaded or received in its normalized form, except that we renormalized the Voineagu et al. dataset to include more probes. Our renormalized version of the Voineagu et al. dataset was the same except that we eliminated probes that did not have significant expression (detection *p* < 0.05) in at least half of the autism or control samples, rather than half of the samples overall. All three studies for the microarray datasets used log2 transformation and quantile normalization. Voineagu et al. showed that all samples met quality control parameters, specifically, if the interarray Pearson correlation was not greater than 0.85 and if the array was an outlier in hierarchical clustering [17]. Chow et al. similarly eliminated samples based on interarray correlation but also used ComBat [26] to correct for batch effects. The Chow et al. data preprocessing pipeline is described in greater detail separately [27]. After data processing, the Voineagu et al. dataset had 12,632 probes and 10,901 unique Entrez gene identifiers. Chow et al. and Garbett et al. did not eliminate probes, resulting in 18,491 and 20,750 Entrez genes, respectively. All other conversion between gene or probe identifiers were performed using the R package biomaRt [28]. Other than in the Voineagu et al. dataset, we used all available genes in the arrays of these datasets.

### Statistical analysis

Using Ensembl gene identifiers from Parikshak et al., functional annotation clustering of gene sets was performed in DAVID (Database for Annotation, Visualization, and Integrated Discovery) [29] using all available gene pathways, including all Gene Ontology (GO) [30] gene sets, and default parameters of DAVID, including medium classification stringency [29]. The background set of genes in DAVID analysis was the list of all protein-coding genes in the Parikshak et al. RNA-seq dataset, and for the Voineagu et al. study, the background was the list of unique Entrez identifiers in the dataset. All other statistical analyses were performed using R 3.3.2. We performed differential gene expression analysis using the Bioconductor package limma with an empirical Bayes adjustment [31], and we adjusted for RIN, PMI, age, sex, and cortical location (temporal vs. frontal). *p* values were corrected for multiple testing using the Benjamini-Hochberg method [32]. For DAVID analysis of the differentially expressed genes from the Voineagu et al. data, if multiple Entrez identifiers mapped to the same Illumina probe, which was true for nine of the downregulated probes and six of the upregulated probes, then a single Entrez identifier was chosen at random to avoid over-representing a single genomic feature. Additionally, all duplicate Entrez identifiers, which was true for 15 of the downregulated genes and five of the upregulated genes, were removed prior to DAVID analysis. In validation analyses, we used all available genes from a pre-specified pathway. We then calculated mean expression of these genes and determined a signature’s differential expression using a *t* test. Heatmaps were generated using the made4 package [33], with Euclidian distance as the distance function.

## Results

### Discovery of a mitochondrial pathway downregulated in autism cerebral cortex

We set out to discover other biological processes that were not previously reported to be differentially regulated in the Parikshak et al. [19] or Voineagu et al. [17] studies. Parikshak et al. provided a list of genes that are differentially expressed between autism and control cerebral cortex, adjusted for RNA quality, age, sex, brain region, and batch. These genes were up- or downregulated in autism cerebral cortex compared to control (see Additional file 3: Table S2 from Parikshak et al. [19]).

We performed separate DAVID functional annotation clustering analyses for the up- and downregulated genes from Parikshak et al. For the upregulated genes, few gene sets were significantly enriched after Benjamini-Hochberg adjustment [32] (Additional file 3: Table S2). However, each of the top 2 clusters among the downregulated genes included multiple gene sets that were significantly enriched after Benjamini-Hochberg adjustment (Additional file 4: Table S3). For the downregulated genes, the gene set cluster with the highest score was largely related to synapse function and the gene set cluster with the second highest score was related to mitochondrial function. In both the Voineagu et al. [17] and Parikshak et al. [19] studies, the authors described differential expression of genes related to synaptic function. Given that a mitochondrial pathway had not previously been reported by Voineagu et al. or Parikshak et al., we decided to focus on this next. We defined the “synapse pathway” as the downregulated genes that overlapped with the UniProt keyword Synapse and the “mitochondria pathway” as those that overlapped with the GO term “Mitochondrion” (Additional file 5: Table S4). To ensure that the mitochondria pathway did not describe synaptic function, we excluded from the mitochondria pathway any genes that were in the synapse pathway or in a related gene set, module M12 from Voineagu et al. [17]

### Validation of the mitochondria pathway’s downregulation

**Fig. 1:**
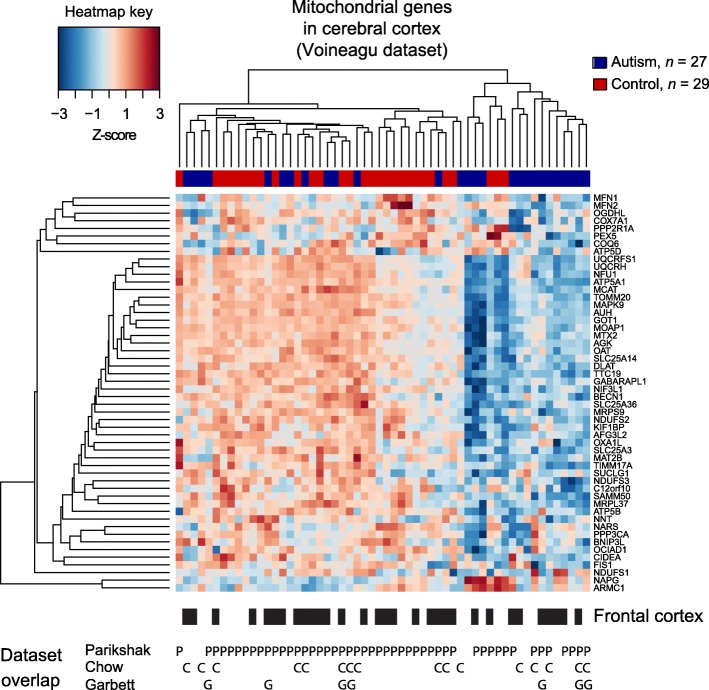
Heatmap of mitochondrial genes in the Voineagu et al. microarray dataset. The rows are genes and the columns are subjects; the top vertical bar shows whether a subject was from autism (blue) or control (red). Generally, lower gene expression (blue in heatmap) maps onto the autism participants (blue in the vertical bar at top of map). Intensity of color is determined by a *Z*-score normalized by gene. Below the heatmap is indicated whether the sample is from frontal cortex (black bar) or temporal cortex (blank space). Also shown below the heatmap is the overlap of each sample with other study datasets, using the first letter of each study

**Fig. 2:**
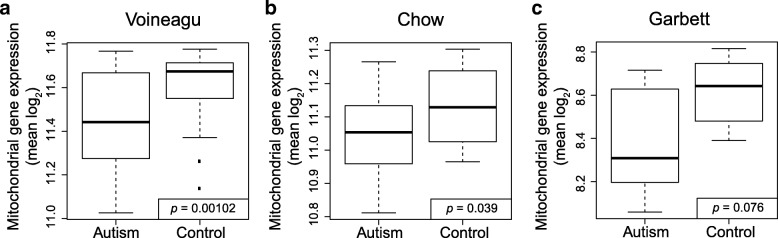
**a**–**c** Boxplots of the mean mitochondria pathway gene expression across the three indicated microarray datasets

To exclude the possibility that the mitochondria pathway’s downregulation was unique to the Parikshak et al. study, we next validated this downregulation in other genomic datasets. We used three microarray studies for validation (Additional file 1: Figure S1 and Additional file 2: Table S1). Because the Voineagu et al. study’s subjects were nearly all in the Parikshak et al., it served largely as technical validation (see Fig. 1 for a heatmap of the genes in the Voineagu et al. dataset and Additional file 6: Figure S2 for similar heatmaps regarding the Chow et al. and Garbett et al. datasets). The mean expression of these mitochondria pathway genes was downregulated in the Voineagu et al. dataset (*t* test *p* = 0.001), Chow et al. dataset (*p* = 0.039), and Garbett et al. dataset (*p* = 0.076) (Fig. 2). To ensure that the downregulation of the mitochondria pathway in the Voineagu et al. dataset was not due to confounders, we also did a separate logistic regression adjusting for RIN, PMI, age, sex, and cortical location (temporal vs. frontal) and found that the mitochondria pathway was still downregulated in autism (*p* = 0.0054). It was also downregulated in a similar multivariate analysis after limiting the dataset only to frontal cortex (*p* = 0.041) or temporal cortex (*p* = 0.057). While the mitochondria pathway was associated with autism, it was not associated with seizures, speech delay, motor delay, or global functioning in the Voineagu et al. dataset (*p* > 0.3 for each comparison).

Because these studies’ subjects overlapped, we did a separate validation analysis of the Chow et al. and Garbett et al. datasets after removing all but the subjects unique to these studies. The mitochondria pathway was still downregulated in these analyses, although the *p* values were not significant (*p* = 0.12 for the Chow et al. dataset and *p* = 0.33 for the Garbett et al. dataset), likely because of reduced sample sizes (*n* = 20 for the Chow et al. dataset and *n* = 7 for the Garbett et al. dataset).

### Genes associated with mitochondrial function and synaptic function strongly correlate

**Fig. 3:**
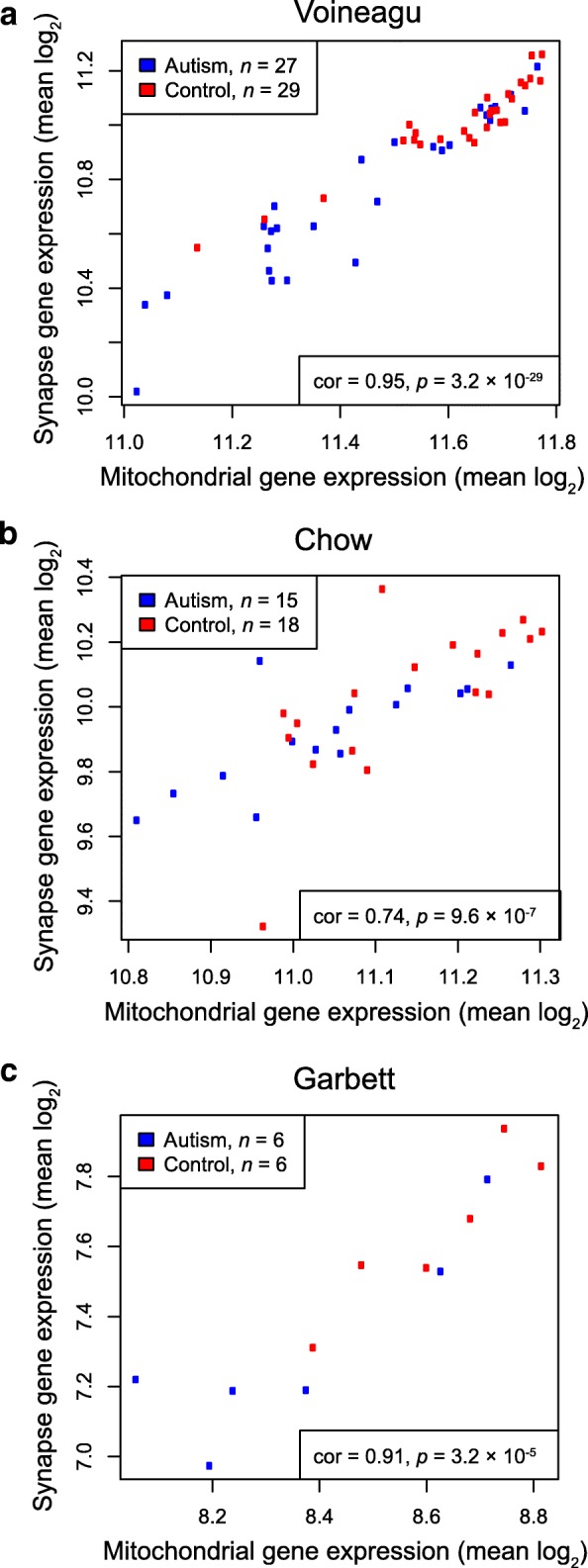
Mean expression of the mitochondria pathway genes plotted against mean expression of the synapse pathway genes for the three indicated microarray datasets. **a**–**c** Mitochondrial gene expression and synapse gene expression were correlated in the Voineagu et al. (**a**), Chow et al. (**b**), and Garbett et al. (**c**) datasets. Correlation coefficients (cor) are shown and reflect a very high level of correlation

In our reanalysis of the Parikshak et al. genes, the synapse-related gene sets were the most strongly enriched in those downregulated in autism, so we next determined the relationship between those synapse-related gene sets and the mitochondria pathway. Across all three microarray datasets, these two pathways had strong Pearson correlation (Fig. 3). To exclude the possibility that such correlation was common, we also used the Voineagu et al. dataset to randomly sample without replacement 10,000 gene sets of similar size to the synapse pathway, and we found that the mitochondria pathway had greater correlation with the synapse pathway than all but 0.37% of random gene sets. For a specific example of correlated genes, in the Voineagu et al. dataset, *GABRA1*, which codes for a gamma-aminobutyric acid (GABA) receptor subunit, and *ATP5A1*, which codes for an ATP synthase subunit, were strongly correlated (correlation = 0.876).

### Alternative gene set enrichment confirms downregulation of mitochondria-associated genes

Given that the mitochondria pathway was among the most enriched in the Parikshak et al. downregulated genes, we did a separate analysis to confirm the importance of this pathway in autism cerebral cortex. Using the Voineagu et al. dataset, we performed a differential gene expression analysis using limma [31] between autism and control cerebral cortex, adjusting for RIN, PMI, age, sex, and cortical location (temporal vs. frontal). This produced 185 upregulated and 247 downregulated unique genes (Additional file 7: Table S5). Given that several individuals were represented twice in this analysis (both for frontal and temporal cortexes), we also did separate analyses using only temporal or frontal cortex samples (Additional file 7: Table S5). We did not adjust for multiple testing in these cortex-specific analyses because no genes were differentially expressed after Benjamini-Hochberg adjustment in these limited samples. At least 84% of the original up- and downregulated genes were in the respective cortex-specific up- or downregulated genes, suggesting broad similarity in these three differential expression analyses.

We next performed DAVID functional annotation clustering of the original up- and downregulated genes. The upregulated genes were enriched in only one gene set (Additional file 8: Table S6), but the downregulated genes were enriched in several gene sets, and all of the top 5 highest scoring gene set clusters were related to mitochondria (Additional file 9: Table S7).

The Voineagu et al. study limited their differential gene expression analysis to genes that had a fold change > 1.3. The mitochondria pathway may have previously gone unreported in that study because in the Voineagu et al. dataset, the mitochondria pathway genes had on average 1.13-fold change in gene expression while the synapse pathway genes had 1.20-fold change. Similarly, in the Parikshak et al. study, the synapse pathway genes showed on average 1.25-fold change while the mitochondria pathway genes showed 1.16-fold change. Thus, the enrichment may not have been detected because of the greater fold change for the synapse genes.

Finally, we also observed that the GABA-related genes in particular were differentially expressed. The synapse pathway included genes coding for two different GABA receptor subunits, and the gene coding for parvalbumin, which is a marker of inhibitory interneurons [34], was the most strongly downregulated gene. The gene coding for parvalbumin was also the most strongly downregulated gene in the Parikshak et al. study [19].

## Discussion

We have conducted a reanalysis of autism and control post-mortem brain gene expression using a recent RNA-seq study [19] and three other similar gene expression studies [16–18]. We discovered that genes related to mitochondria are significantly downregulated in autism brains relative to control. Abnormalities related to mitochondria have been implicated in autism pathogenesis through several lines of evidence, such as over-representation of mitochondrial disease in ASD patients and elevation of biomarkers of metabolism such as lactate and pyruvate [22]. Further, genes for select electron transport chain complexes have been shown to be lowly expressed in the cortex of children with autism [35].

We also observed that this mitochondria pathway gene expression correlated strongly with that of a synapse pathway, suggesting a common pathophysiology. Consistently, Gandal et al. recently described a gene module related to synaptic transmission and mitochondria that was downregulated in both autism and schizophrenia [36]. Schizophrenia has also previously been shown to have decreased expression of mitochondria-related genes [24].

In our study, we noted other similarities to schizophrenia, as well. For example, we particularly noted that genes related to inhibitory interneurons were downregulated. In prior studies, *GAD1* and *GAD2* have been shown to be reduced in parietal and cerebellar cortex in autism [37] and GABA receptor density is reduced in post-mortem autism cerebral cortex [38]. Similar inhibitory interneuron gene alterations are seen in the cerebral cortex in schizophrenia [39]. The reason for common downregulation of inhibitory interneuron and mitochondrial genes in autism and schizophrenia is unclear. However, it is noted that both conditions are also associated with gene-by-environment interactions related to the immune system, suggesting a similar pathophysiology [40]. The immune system’s role is evidenced by each condition’s association with maternal immune activiation during pregnancy [41, 42], as well as with genetic variation in major histocompatibility complex genes [43, 44].

Because we have not explored protein or functional analyses, we cannot discern whether these gene expression changes are part of the primary pathology or secondary pathology or both. However, in vitro experiments have shown a close interplay between mitochondria and synapse regulation. For example, Li et al. showed that GTPases that control mitochondrial fission and fusion also regulate synapse plasticity and density [45]. These researchers further showed that increased neuronal activity increased mitochondrial fission in a neuron while decreased activity increased fusion, suggesting a mitochondrial response to neuronal energy needs. For autism, primary synaptic dysregulation could result in reduced neuronal energy demand and thus mitochondrial activity. Alternatively, several studies, including those that report gene mutations or susceptibility variants in mitochondrial genes [46, 47], support the notion that primary mitochondrial defects may occur in autism. Regardless, abnormalities in mitochondria are a feature of synaptic gene dysregulation in idiopathic autism and deserve additional study. A pertinent question that results is whether autism symptoms would be responsive to medicines or supplements that are used in treatment of primary mitochondrial disease. This hypothesis has been tested in small studies [48], but further studies may be warranted, particularly after peripheral biomarkers become available for stratifying patients into groupings that may be more amenable to these treatments.

Several other factors might affect differential expression. To account for possible confounders of the association between autism and gene pathways, we adjusted for RIN, PMI, sex, age, and cortical region in our analyses. However, some variables were not available for multivariate analysis, including those related to treatment, lifestyle, and other technical confounders. However, given that the analyses validated across datasets, the pathway results are robust, and other possible confounders are unlikely to alter interpretation of these pathways’ associations with autism.

Although the magnitudes of the expression changes of each pathway were relatively small, small-magnitude gene expression differences can still have profound effects, a finding seen in other psychiatric conditions, including schizophrenia [49, 50]. Additionally, because each cortical sample is from a heterogeneous cell population, small changes may also represent dilution of a single cell type’s gene expression changes. In our study, gene expression changes likely reflect neurons, given the observed correlations with genes related to synaptic and axonal function.

## Conclusions

ASDs are a heterogeneous group of diseases with many proposed pathophysiological mechanisms. We have used multiple genomic datasets to investigate the pathophysiology of autism by analyzing gene expression patterns. Our study provides support for hypotheses related to mitochondrial dysfunction. Additionally, we provide strong evidence for the coordinated dysregulation of synaptic and mitochondrial function. With gene expression alone and without protein or functional assays, it is not clear whether the synapse and mitochondria pathways are downstream of the biology of interest or primary processes. Thus, these coordinated gene pathways should be kept in mind as we move forward with dissecting molecular networks at the cellular and circuit level in experimental systems.

## Additional files

Additional file 1: **Figure S1.** Venn diagram depicting the overlap of participants between the RNA-seq dataset and the three microarray datasets analyzed in this study. See the “Participants” section under the “Methods” section for more information on each study. Both autism and control subjects are included in the Venn diagram. (PDF 176 kb)

Additional file 2: **Table S1.** Properties of the Parikshak, Voineagu, Chow, and Garbett datasets. Table depicting the properties of the datasets analyzed in this study. Properties listed include sample size, number of features in the dataset, brain region, age range, gender, PMI range, RIN cutoff, and the source of the cases. (XLSX 12 kb)

Additional file 3: **Table S2.** DAVID functional annotation clustering analysis of Parikshak et al. upregulated genes. Using the genes upregulated in autism cerebral cortex from Parikshak et al., DAVID functional annotation clustering was performed to generate groups of enriched gene sets. (XLSX 118 kb)

Additional file 4: **Table S3.** DAVID functional annotation clustering analysis of Parikshak et al. downregulated genes. Using the genes downregulated in autism cerebral cortex from Parikshak et al., DAVID functional annotation clustering was performed to generate groups of enriched gene sets. (XLSX 134 kb)

Additional file 5: **Table S4.** Synapse pathway and mitochondria pathway genes. The mitochondria pathway genes were downregulated in autism cerebral cortex in Parikshak et al. and were members of the GO “Mitochondrion” term. The synapse pathway genes were also downregulated in Parikshak et al. and were members of the UniProt “Synapse” term. All genes in the synapse pathway and in the related “M12” module from Voineagu et al. [17] were excluded from the mitochondria pathway. (XLSX 16 kb)

Additional file 6: **Figure S2.** Heatmaps of mitochondrial genes in the Chow et al. and Garbett et al. microarray datasets. The rows are genes and the columns are subjects; the top vertical bar shows whether a subject was from autism (*blue*) or control (*red*). Generally, lower gene expression (*blue in heatmap*) maps onto the autism participants (*blue in the vertical bar at top of map*). Intensity of color is determined by a *Z*-score normalized by gene. Shown below the heatmap is the overlap of each sample with other study datasets, using the first letter of each study. (PDF 577 kb)

Additional file 7: **Table S5.** Differential expression analysis between autism and control cerebral cortex in the Voineagu et al. dataset. Table of differential expression analysis between autism and control cerebral cortex in the Voineagu et al. dataset, adjusted for RIN, PMI, age, sex, and cortical location (temporal vs. frontal). The analysis was performed using the Bioconductor package limma and adjusted for multiple testing using the Benjamini-Hochberg method. Because several individuals were included twice (for both frontal and temporal cortexes) in this analysis, the analysis was also redone limiting samples only to frontal or temporal cortex. Note that the *p* values in the cortex-specific analyses are not adjusted for multiple testing. (XLSX 265 kb)

Additional file 8: **Table S6.** DAVID functional annotation clustering analysis of Voineagu et al. upregulated genes. Using the genes upregulated in autism cerebral cortex from Voineagu et al., DAVID functional annotation clustering was performed to generate groups of enriched gene sets. (XLSX 48 kb)

Additional file 9: **Table S7.** DAVID functional annotation clustering analysis of Voineagu et al. downregulated genes. Using the genes downregulated in autism cerebral cortex from Voineagu et al., DAVID functional annotation clustering was performed to generate groups of enriched gene sets. The top 5 clusters had several gene sets related to mitochondrial function. (XLSX 49 kb)

## Abbreviations

ASD: Autism spectrum disorder
DAVID: Database for Annotation, Visualization, and Integrated Discovery
GABA: Gamma-aminobutyric acid
GEO: Gene Expression Omnibus
GO: Gene Ontology
PMI: Post-mortem interval
RIN: RNA integrity number
