# Supplementary figures and images for: Strong correlation of downregulated genes related to synaptic transmission and mitochondria in post-mortem autism cerebral cortex
Source: J Neurodev Disord. 2018 Jun 1;10:18. doi: 10.1186/s11689-018-9237-x (PMC5984825; doi:10.1186/s11689-018-9237-x)

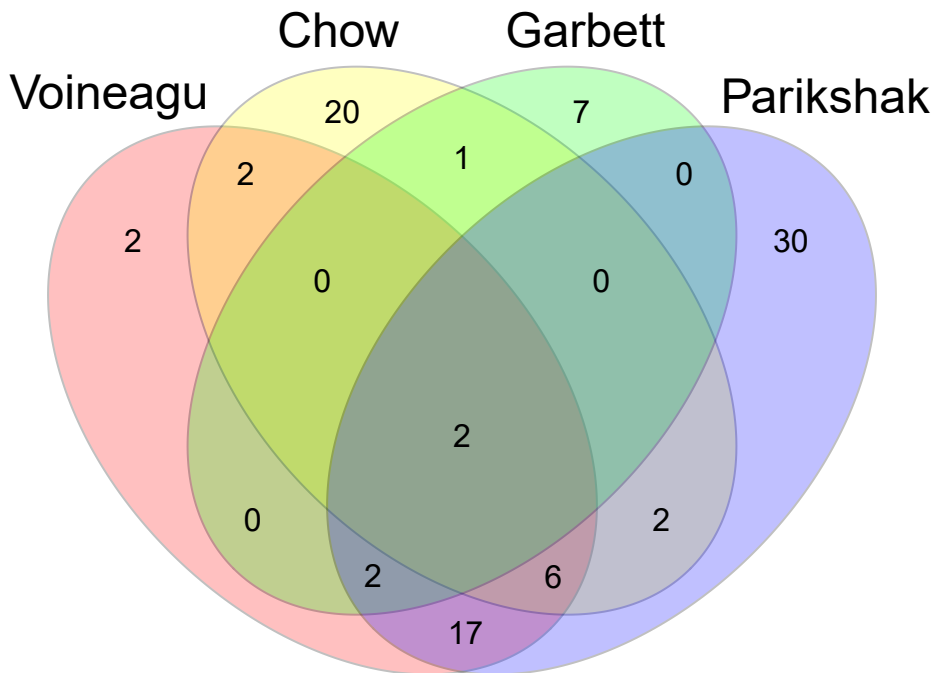

Supplement: Supplementary file 1 — Figure S1. Venn diagram depicting the overlap of participants between the RNA-seq dataset and the three microarray datasets analyzed in this study. See the “Participants” section under the “Methods” section for more information on each study. Both autism and control subjects are included in the Venn diagram. (PDF 176 kb) [file 11689_2018_9237_MOESM1_ESM.pdf]

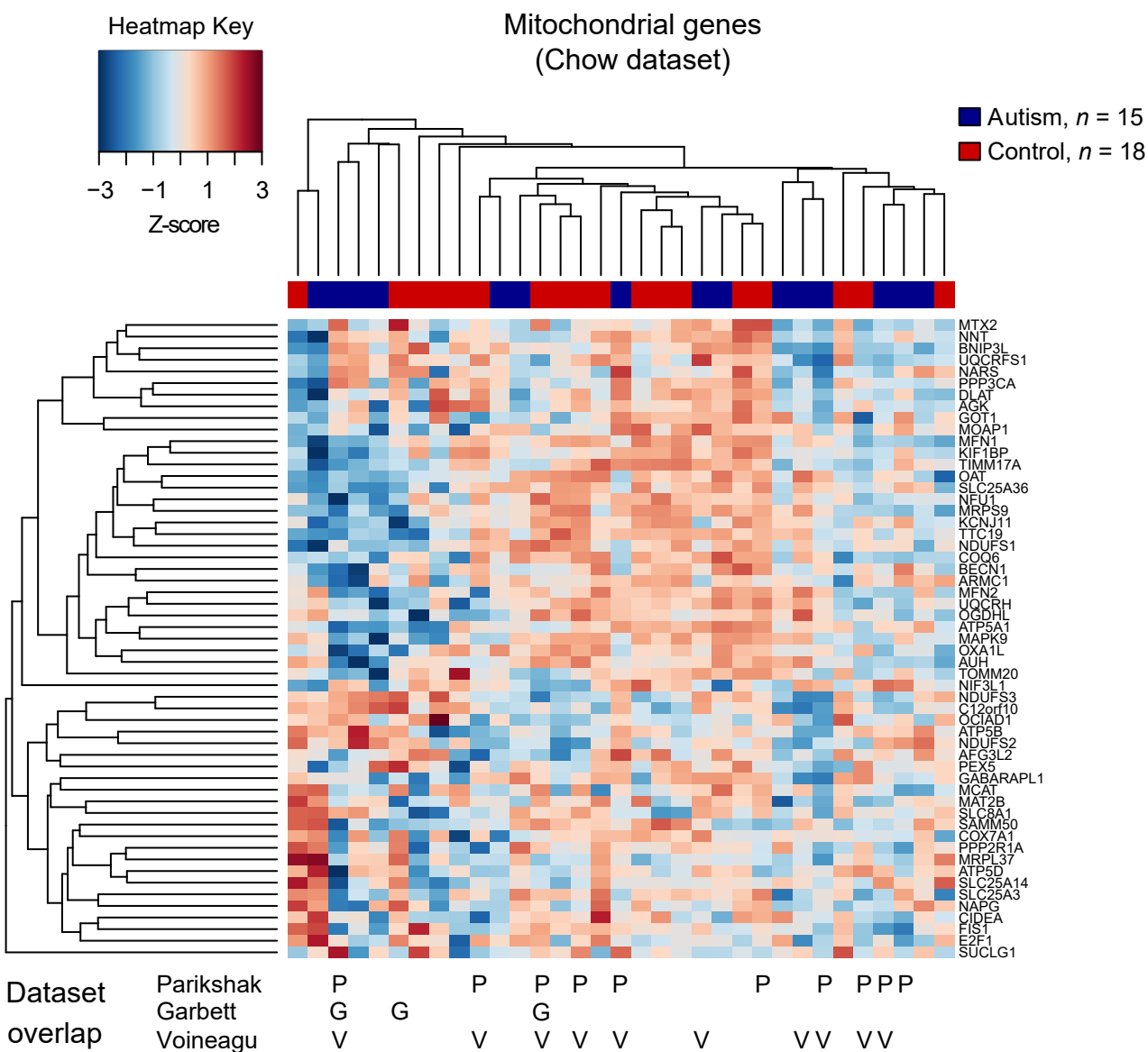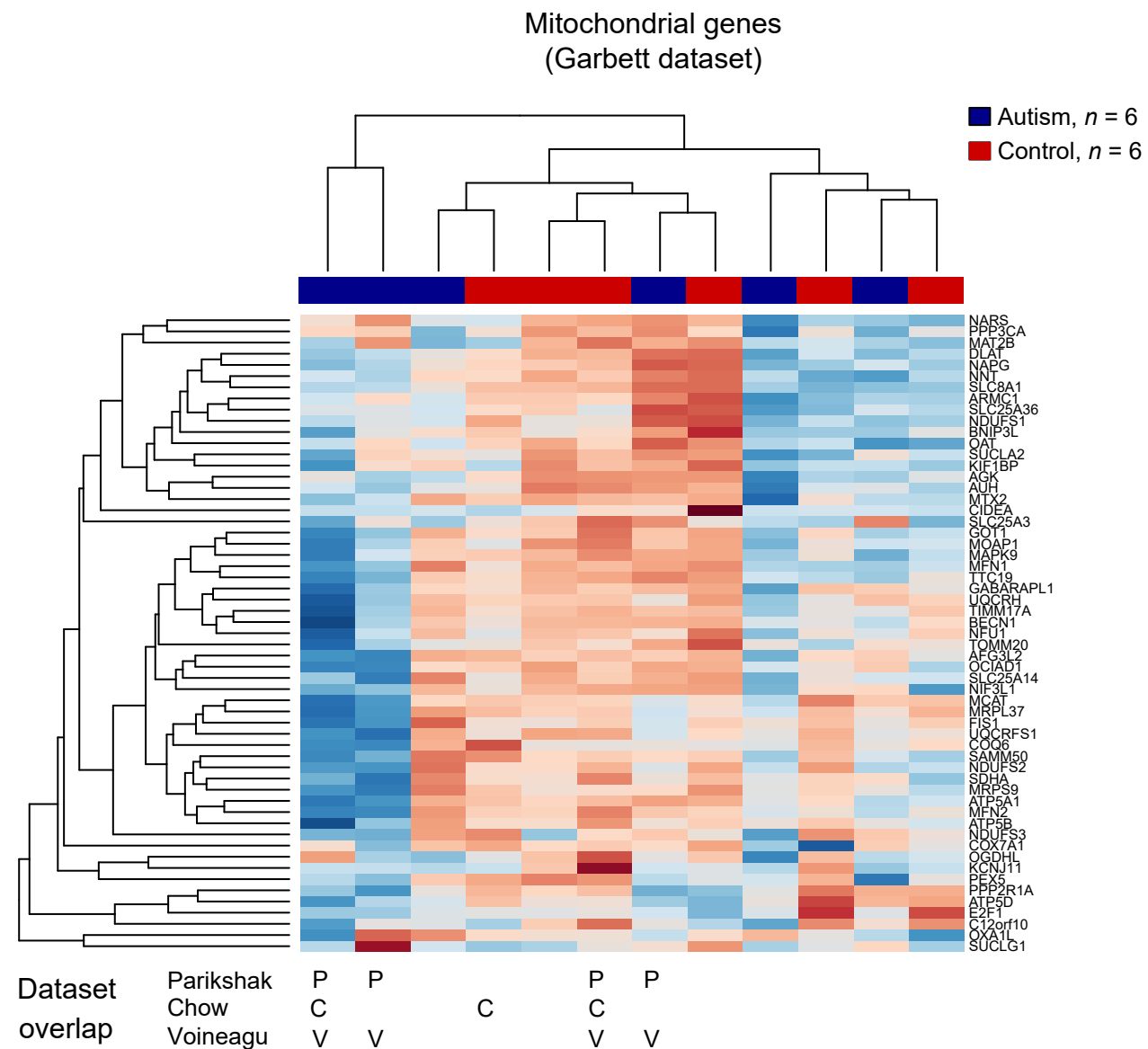

Supplement: Supplementary file 6 — Figure S2. Heatmaps of mitochondrial genes in the Chow et al. and Garbett et al. microarray datasets. The rows are genes and the columns are subjects; the top vertical bar shows whether a subject was from autism (blue) or control (red). Generally, lower gene expression (blue in heatmap) maps onto the autism participants (blue in the vertical bar at top of map). Intensity of color is determined by a Z-score normalized by gene. Shown below the heatmap is the overlap of each sample with other study datasets, using the first letter of each study. (PDF 577 kb) [file 11689_2018_9237_MOESM6_ESM.pdf]
